# Inadequate detection of the FSHR complicates future research on extragonadal FSHR localization

**DOI:** 10.3389/fendo.2023.1095031

**Published:** 2023-02-16

**Authors:** Victoria N. Tedjawirja, Gerrit K. J. Hooijer, C. Dilara Savci-Heijink, Kristina Kovac, Ron Balm, Vivian de Waard

**Affiliations:** ^1^ Department of Surgery, Amsterdam Cardiovascular Sciences, Amsterdam University Medical Centre (UMC), University of Amsterdam, Amsterdam, Netherlands; ^2^ Department of Pathology, Amsterdam University Medical Centre (UMC), University of Amsterdam, Amsterdam, Netherlands; ^3^ Department of Medical Biochemistry, Amsterdam Cardiovascular Sciences, Amsterdam University Medical Centre (UMC), University of Amsterdam, Amsterdam, Netherlands

**Keywords:** follicle stimulating hormone receptor, extragonadal cells, antibodies, immunohistochemistry, control

## Abstract

**Introduction:**

Recently, follicle stimulating hormone (FSH) through interaction with its receptor (FSHR) has been proposed to play a role in postmenopausal osteoporosis and cardiovascular disease, rather than the loss of estrogen. To explore this hypothesis, unravelling which cells express extragonadal FSHR on protein level is key.

**Methods:**

We used two commercial anti-FSHR antibodies and validated them by performing immunohistochemistry on positive (ovary, testis) and negative controls (skin).

**Results:**

The monoclonal anti-FSHR antibody could not identify the FSHR in ovary or testis. The polyclonal anti-FSHR antibody stained the granulosa cells (ovary) and Sertoli cells (testis), yet there was equally intense staining of other cells/extracellular matrix. Furthermore, the polyclonal anti-FSHR antibody also stained skin tissue extensively, suggesting that the antibody stains more than just FSHR.

**Discussion:**

The findings in this study may add accuracy to literature on extragonadal FSHR localization and warrants attention to the use of inadequate anti-FSHR antibodies to value the potential role of FSH/FSHR in postmenopausal disease.

## Introduction

Extragonadal sites with a functional FSH receptor (FSHR) were identified in hepatocytes, adipocytes, vascular endothelial cells (EC), monocytes/macrophages, and osteoclasts (1–4). This provoked the hypothesis that the elevated follicle-stimulating hormone (FSH) levels across the menopausal transition, rather than the decline in estrogen levels, could play a role in the development of postmenopausal osteoporosis and cardiovascular disease (CVD) for which encouraging data have been published (1–4). Similarly, the enhanced development of abdominal aortic aneurysm (AAA), a dilatation of the aorta which can be life-threatening upon rupture (5), in postmenopausal women has been attributed to the decrease of serum estrogen (6, 7). However, the effect of hormonal replacement therapy on AAA in postmenopausal women was inconclusive (8–10) and perhaps another hormonal alteration during the menopause should be considered. We hypothesized that FSH may play a direct role in the onset/progression of AAA in postmenopausal women, potentially through the activation of EC and monocytes/macrophages. In our attempt to unravel which cells within the aorta express the FSHR on their cell surface, we used two different commercial anti-FSHR antibodies. However, we faced an unexpected lack of staining or unspecific staining, which we think is important to share.

## Methods

### Materials

Human premenopausal ovary, testis, and skin tissue without any abnormalities were obtained from the Department of Pathology [Amsterdam University Medical Centre (Amsterdam UMC)] for the current study. Tissue specimens were retrospectively collected from the Biobank Tissue Archive Pathology (2015_081), which was reviewed and approved by the Committee Review Biobanks of the Amsterdam UMC. All materials were coded and handled in accordance with the national ethical guidelines (“Code of Conduct for Health Research” developed by the Dutch Committee on Regulation of Health Research).

### Immunohistochemistry

Of the multiple immunohistochemical (IHC) stainings conducted by the departments Medical Biochemistry and Pathology, the most promising procedure is outlined.

IHC was conducted on ovary and testis tissues that indisputably should express the FSHR (in granulosa and Sertoli cells, respectively) to validate the purchased antibodies. The formalin-fixed paraffin-embedded (FFPE) tissues were cut into 4-µm sections, mounted on coated slides, and dried overnight (37°C). The slides were deparaffinized with xylene (3 x 5min) and rehydrated with ethanol (100%, 100%, 96%, and 70%, 20 dips each). Endogenous peroxidase was quenched with 0.5% H_2_O_2_ in methanol (15min). Then, we assessed the most optimal antigen retrieval step by rinsing the slides in demineralized water and using three different solutions. Antigen retrieval with citric acid buffer pH 6.0 and Tris-EDTA buffer pH 9.0 were performed in a pressure cooker (20 min, 120°C). The third method was performed in 0.25% pepsin in 0.01 M HCl (10 min, 37°C). Hereafter, the slides were washed in phosphate-buffered saline with Tween (PBST) (1 x 3min). The sections were incubated with either the primary mouse monoclonal antibody directed against the human FSHR (clone FSHR/1400, NSJ Bioreagents; unclear which part of the FSHR it recognizes) or the primary rabbit anti-FSHR polyclonal antibody (MBS178821, MyBioSource; raised against the C18-N187 peptide sequence) in Normal Antibody Diluent (ABD999, Immunologic). After washing with PBST (3x3min), the sections were incubated with the specific polymers Brightvision poly-HRP-anti mouse Ig or Brightvision poly-HRP-anti rabbit Ig (DPVR110HRP, Immunologic), respectively, as a ‘secondary antibody’. Bright DAB (BS04-110, Immunologic) was used for visualization (8 min). The ovary tissue was incubated with inhibin-α (IC25-4065, Instruchemie) (1:25, 32min) with the Ventana Benchmark Ultra Instrument and visualized with the Ventana’s OptiView DAB IHC detection kit. Skin tissue was pre-treated in Tris-EDTA buffer pH 9.0 and incubated with Cytokeratin17 (Ks.17.E3, NBP2-29421, Novus) (1:100, 60min). Hereafter, the sections were washed in running tap water, rinsed in demineralized water, counterstained with hematoxylin 1:5 (5min), and, for color development, washed in running tap water (5 min) to visualize cellular nuclei. The slides were dried at 59°C, dipped in xylene, and mounted in Pertex (00801, Histolab).

Skin tissue, not known to have FSHR expression, was used as negative control for tissue specificity. As negative control for unexpected staining by the Brightvision polymers as ‘secondary antibody’, sections of ovary, testis, and skin were incubated as described above, but omitting the primary anti-FSHR antibodies.

## Results

**Figure 1:**
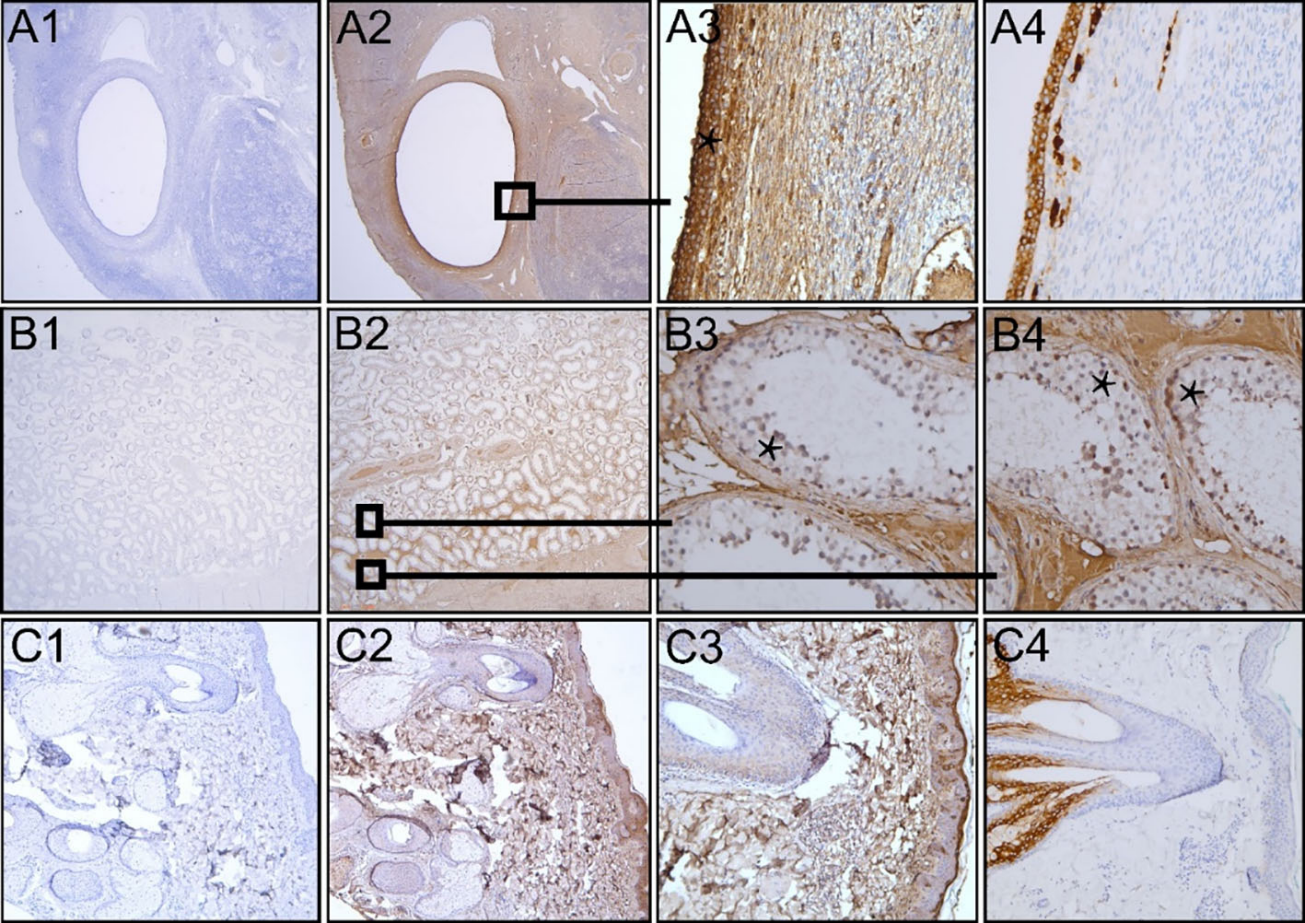
Immunohistochemical analysis of FFPE human premenopausal ovary, testis and skin tissue (**A–C**, respectively). Polyclonal anti-FSHR antibody was ommitted (A1, B1 and C1) or the sections were incubated with the antibody (A2-3, B2-4, C2-3 in 1:500 dilution). Sections that were not incubated with the anti-FSHR antibody only showed blue nuclear staining. The asterisks show FSHR positive (red/brown) granulosa cells (A3) and possibly FSHR positive Sertoli cells among other positive cells in the seminiferous tubules of the testis (B3, B4). Staining inhibin in ovary tissue reveals how specific granulosa staining can be (A4). Staining of skin tissue with anti-cytokeratin 17 antibody shows specific staining of a hair follicle with its sebaceous gland (C4), as opposed to the aspecific staining throughout the skin tissue section by the anti-FSHR antibody. Magnification: Panels A1-2, B1-2, and C1-2 12.5x; Panels A3-4, B3-4, and C3-4 200x; Panel C4 100x.

Incubation of the ovary tissue with the monoclonal anti-FSHR antibody (1:100, 60 min/overnight, 37°C) revealed no staining of the granulosa cells with any of the three antigen retrieval methods. Subsequently, to enhance the chance of FSHR staining, we used the polyclonal anti-FSHR antibody as it may recognize multiple epitopes (11). From all three antigen retrieval methods, boiling the sections in citric acid buffer pH 6.0 gave the most optimal staining (1:500, overnight, 4°C). Yet, in addition to granulosa cell staining, there was intense non-specific background staining. To diminish this, the antibody was further diluted (1:750, 1:1000, 1:1500), yet this reduced the entire staining instead of enhancing the specific/non-specific ratio. Granulosa cell staining disappeared at a dilution of 1:750 and beyond. We thus performed the FSHR staining on ovary, testis, and skin tissue with the polyclonal anti-FSHR antibody (1:500, citric acid buffer, overnight, 4°C) (**Figures 1A2-3, B2-4, C2-3**). The granulosa cells stained FSHR positive as expected (asterisk **Figure 1A3**). However, the severe additional non-specific cellular and extracellular matrix staining makes it difficult to trust the specificity of the antibody, especially when studying the extragonadal tissue, where one does not know which cells should be positive for the FSHR. Ovary tissue staining with an anti-inhibin antibody shows how granulosa cell staining could be when it is specific (**Figure 1A4**). Similar findings were obtained in testis tissue, where Sertoli cells were probably FSHR positive among additional positive cells in the seminiferous tubules (asterisks **Figure 1B3-4**). However, since the polyclonal antibody also stained the stroma and Leydig cells, the reliability of this antibody is questioned. Application of solely the secondary antibodies revealed no staining in ovary, testis, and skin tissue sections, revealing that all staining is caused by the anti-FSHR polyclonal antibody (**Figures 1A1, B1, C1**). In skin tissue, the monoclonal anti-FSHR antibody showed no staining at all; however, the polyclonal anti-FSHR antibody gave positive staining of the stroma, adipocytes, and hair follicles, which indicates abundant non-specific staining (**Figure 1C2-3**). While adipocytes have been reported to be able to express the FSHR (12), the stroma and hair follicles should not. Specific staining in skin tissue is demonstrated with the anti-cytokeratin 17 antibody, which shows a positive hair follicle and sebaceous gland, as expected (**Figure 1C4**). In conclusion, the two anti-FSHR antibodies could either not detect the FSHR or recognized more than just the FSHR.

## Discussion

We aimed to identify FSHR-bearing cells within the aortic wall, through which FSH may affect AAA onset/progression in postmenopausal women. Exploring this hypothesis, we conducted IHC staining with two anti-FSHR antibodies on positive/negative control tissues for validation. The monoclonal anti-FSHR antibody did not perform in its ability to identify the FSHR in ovary or testis. The polyclonal anti-FSHR antibody stained the granulosa cells and possibly Sertoli cells, yet the additional staining of the other cells/extracellular matrix was equally intense. Since also in the skin tissue, which is devoid of known FSHR expression, staining was observed, we doubt the specificity of the polyclonal antibody. With both antibodies we had observed occasional positive macrophages, EC and smooth muscle cells in our aorta sections (obtained from anonymous donors, data not shown), which is uncertain if this is true FSHR localization and thus relevant. The reason to bring our findings to light is to add accuracy to literature, as the use of non-specific anti-FSHR staining jeopardizes the interpretation of FSHR localization in extragonadal tissues.

Various anti-FSHR antibodies have been used to study extragonadal FSHR protein expression and localization (1–4, 12–17). Chrusciel et al. summarized some commonly (and currently unavailable) used antibodies in extragonadal FSHR research (18). Hybridoma FSH323, validated by positive IHC staining of granulosa and Sertoli cells, is commercially unavailable (13, 14, 17). Interestingly, two groups that used FSH323-derived antibodies showed positive controls in their manuscript but obtained different results. Stilley et al. detected positive staining in human umbilical cord venous EC (14), whereas Stelmaszewska et al. did not and also did not find FSHR mRNA transcripts in EC (17). Another anti-FSHR antibody from Abcam used in extragonadal FSHR research detected the presence of FSHR in human hepatocytes (3) and adipose tissue (12), which was validated using negative/positive IHC controls. However, without stating the catalogue number, it is unclear which antibody was used. Perhaps it was the unavailable rabbit polyclonal antibody ab150557, shown to be specific to FSHR and raised against an unspecified N-terminal peptide, that was expected to identify the canonical FSHR and short variant of FSHR (19), or three other currently available anti-FSHR antibodies by Abcam (1): ab113421 (generated against amino acid sequence 278-327 of the extracellular domain just prior to the transmembrane domain), (2) ab137695 (recognizing the cytoplasmic C-terminal amino acid sequence 631-695), and (3) ab75200 (unclear which part of the FSHR it should recognize). Antibody ab113421 may not recognize the FSHR short variant (lacking exon 9), which is reported in some extragonadal cells (1, 14), because this FSHR isoform misses amino acids 224–285, which in part overlaps with the peptide that is used to generate these antibodies. Although in a comparison paper between various anti-FSHR antibodies, the sc-13935 anti-FSHR antibody from Santa Cruz Biotechnology does not seem to be specific for the human FSHR (20), the antibody has been shown to be specific in another study (19). However, this antibody seems to be no longer available to repeat the experiments.

**Figure 2:**
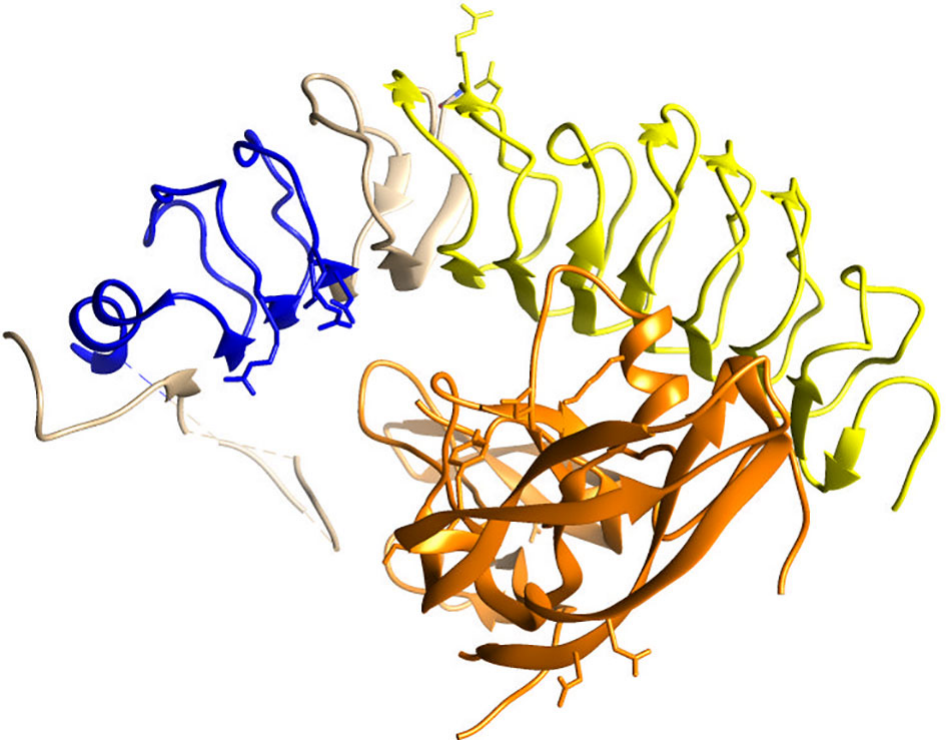
Crystallographic image of the extracellular domain of the FSHR (blue, beige, yellow) folding over its ligand FSH (orange), adapted from Jiang et al. (22). In yellow the beta strands/leucine-rich repeat domains in amino acid stretch C18-N187 are highlighted, against which the polyclonal anti-FSHR antibody MBS178821 is raised. The blue ribbon represent the amino acids that exon 9 codes for, which lacks in the short FSHR variant.

A potential explanation for the polyclonal antibody non-specificity may be that it was tested for Western blotting, while not being optimized for IHC in FFPE tissues. Yet, antibodies validated for Western blotting often perform well in IHC (21). The polyclonal anti-FSHR antibody MBS178821 that we used was raised against the C18-N187 amino acid sequence, and should in theory recognize both the full length and short FSHR variant (**Figure 2**). However, this stretch of amino acids contains multiple beta strands and leucine-rich repeat domains (**Figure 2**) (22), forming a 3D structure, that is quite common in many other proteins (23), including the receptors for the other gonadotropins (22), and possibly explains the additional staining.

Since the concept of extragonadal FSHR expression is interesting to pursue in light of postmenopausal disease, future FSHR research should include control IHC stainings, information on the part against which the FSHR antibody is raised, and the antibodies’ catalogue numbers, which are essential to establish the validity of extragonadal FSHR expression.

## Data availability statement

The original contributions presented in the study are included in the article/supplementary material. Further inquiries can be directed to the corresponding author.

## Ethics statement

The studies involving human participants were reviewed and approved by Committee Review Biobanks of the Amsterdam University Medical Centre. Written informed consent for participation was not required for this study in accordance with the national legislation and the institutional requirements.

## Author contributions

VT and VW contributed to the concept of the work. VT and GH performed the immunohistochemical stainings. GH, CS-H, and VW contributed to the evaluation of the stainings. KK contributed to designing the crystallographic image. VT wrote the first draft of the manuscript and was supported by VW in the writing process. All authors contributed to the article and approved the submitted version.
